# Electrical impedance tomography reveals regional lung dysfunction in adults with preserved ratio impaired spirometry: an observational cross-sectional study

**DOI:** 10.3389/fmed.2025.1678253

**Published:** 2025-10-15

**Authors:** Junsong Zhang, Shuoyao Qu, Mingyi Kong, Liang Han, Xinyu Ti, Zhanqi Zhao

**Affiliations:** ^1^Department of Pulmonary and Critical Care Medicine, Xijing Hospital, Air Force Medical University, Xi'an, China; ^2^School of Biomedical Engineering, Guangzhou Medical University, Guangzhou, China; ^3^Department of Critical Care Medicine, Peking Union Medical College Hospital, Chinese Academy of Medical Sciences, Beijing, China

**Keywords:** PRISm, electrical impedance tomography (EIT), regional lung function, visualize, localize

## Abstract

**Objective:**

Preserved ratio impaired spirometry (PRISm) is a condition characterized by abnormal spirometry results despite a normal forced expiratory volume in the first second (FEV1)/forced vital capacity (FVC) ratio. We hypothesized that electrical impedance tomography (EIT) would detect regional decreases in lung function in subjects with PRISm. The study aimed to explore the regional lung function differences between subjects with preserved ratio impaired spirometry (PRISm) and healthy volunteers.

**Methods:**

In this observational study, subjects with respiratory symptoms visiting Xijing Hospital in May 2024 were rescreened for eligibility. Subjects meeting the PRISm criteria were included in the study. Electrical impedance tomography (EIT) and spirometry were conducted simultaneously. The same number of healthy subjects were studied, matching the gender, height, and weight in frequency. The regional lung function parameters were compared. A *P*-value of < 0.05 was considered statistically significant.

**Results:**

A total of 16 subjects with PRISm, and 16 healthy volunteers were included in the study and evaluated. Functional EIT images provided information about the location of the dysfunctional lung regions. All evaluated EIT-based parameters were worse in the subjects with PRISm. Statistically significant differences were found in the regional obstructive ratio (*rOR*) and the median of the regional time constant τ_med_ (*p* < 0.05). EIT-based parameters did not correlate with spirometry-based parameters, except for spirometry FEV1/FVC, which showed correlations with *rOR* (*r* = −0.53, *p* = 0.03) and τ_med_ (*r* = −0.62, *p* = 0.01) in subjects with PRISm. Both τ_med_ and *rOR* values were quite low in healthy volunteers, whereas substantial changes were observed in subjects with PRISm.

**Conclusion:**

EIT-based regional lung function provides additional information to traditional spirometry, which visualizes and localizes regional lung function defects.

## Introduction

Preserved ratio impaired spirometry (PRISm) is a condition characterized by abnormal spirometry results despite a normal FEV1/FVC ratio (the ratio of the forced expiratory volume in the first second to the forced vital capacity of the lungs). This condition is highly prevalent in several large cross-sectional and longitudinal studies (estimated at 5% to 20% globally) (1), with approximately 10%−40% of patients with PRISm tending to develop chronic obstructive pulmonary disease (COPD) (2), but no tools exist to predict who will deteriorate.

PRISm involves respiratory symptoms and reduced quality of life, despite normal spirometry values at early stages. This means that individuals with PRISm may not be classified as having impaired lung function based on traditional spirometry measures, which rely on the FEV1/FVC ratio and/or FEV1 predicted values. However, previous studies have found an association between decline in quality of life and onset of frequent exacerbations in subjects with normal or PRISm spirometry (3). The pathophysiology of PRISm is not fully understood (1). It may involve abnormalities in lung structure and function that are not detected by traditional spirometry. These abnormalities may include airway inflammation, small airway disease, and/or emphysema. These changes may lead to reduced lung function and increased susceptibility to respiratory infections and exacerbations.

Current treatment for PRISm is limited and often based on symptom management. Because the condition is not well-understood, there is a lack of specific therapies targeted at the underlying pathophysiology. However, lifestyle modifications such as smoking cessation, exercise, and weight management may be beneficial in improving lung function and reducing symptoms. Additionally, inhaled bronchodilators and corticosteroids may be prescribed to help manage symptoms and reduce exacerbations.

Given the high prevalence of PRISm and its association with increased risk of developing COPD, there is a need for more sensitive methods to identify individuals with impaired lung function. Spirometry cannot localize dysfunction, while CT lacks functional data (4). Research is ongoing to develop new diagnostic tools and therapies that can better address the underlying pathophysiology of PRISm and improve outcomes for patients with this condition.

Electrical impedance tomography (EIT) is a non-invasive imaging modality to assess spatial and temporal ventilation distribution (5). A flexible belt with 16–32 electrodes is placed onto the chest. Insensible alternating currents are injected between electrode pairs. Subsequently, the surface voltages are measured and used to reconstruct the electrical impedance changes within the thorax. Lung volume changes are highly correlated with impedance changes (6). Chest EIT applications are mainly in the field of intensive care unit (ICU) [e.g., (7, 8)]. However, since flow is the derivative of volume changes, regional vital capacity and airway flow limitations can be assessed by EIT as well. EIT provides regional lung function information that cannot be captured by other techniques. Such information has been used to demonstrate differences between healthy subjects and patients with asthma (9), COPD (10), cystic fibrosis (11), amyotrophic lateral sclerosis (12), etc. Its application to PRISm, however, has not been well-studied. We hypothesized that EIT would detect regional decreases in lung function in PRISm subjects. The primary aim of this study was to determine whether EIT can characterize distinct patterns of regional lung dysfunction in subjects with PRISm that are not apparent from traditional spirometry. To address this aim, we pursued the following specific objectives:

To compare EIT-derived parameters of regional lung function between PRISm subjects and healthy volunteers.

To visualize the spatial distribution of pulmonary dysfunction in PRISm subjects through functional EIT imaging to identify common patterns.

## Methods

This cross-sectional study was approved by the local ethics committee (No. KY20242044). Informed consent was signed by the subjects before the procedure. In May 2024, subjects with respiratory symptoms who visited our hospital were prospectively screened. Pulmonary Function Test (PFT) was conducted using MasterScreen (Jaeger, CareFusion GmbH, Hoechberg, Germany) according to the American Thoracic Society - European Respiratory Society (ATS-ERS) guidelines (13). EIT was conducted simultaneously with the FVC maneuver (VenTom-200, MidasMED Biomedical Technology, Shenzhen, China). A belt with 16 equidistantly fixed electrodes was placed around the chest in one transverse plane at the level of the 5th intercostal spaces at the parasternal line. For female subjects, if the 5th intercostal space was not accessible, the electrode belt was placed above the breast (~4th intercostal space) (14). Further details of the measurement procedure were described previously (15). The inclusion criteria were: individuals aged between 20 and 80 years; individuals with FEV1/FVC >0.7 after bronchodilator reversibility; individuals with FEV1 % predicted < 80%; and individuals willing to participate in the study. The exclusion criteria were: in hospitalization; malignant tumors, autoimmune diseases, systemic infectious diseases, and dysfunction of vital organs; pleural diseases or chest deformities; any contraindications to pulmonary function test (FVC maneuver); or EIT measurement (e.g., pacemaker, automatic implantable cardioverter defibrillator, and implantable pumps). The same number of healthy subjects were studied, matched by gender, height, and weight frequency. Inclusion criteria for healthy volunteers were: individuals aged between 20 and 80 years; no respiratory symptoms; FEV1/FVC >0.7 and FEV1 % predicted >80%; and willingness to participate in the study.

Subjects' demographics and history of smoking were recorded. EIT data were recorded at 20 Hz and reconstructed with reference to the lowest point of impedance. Regional FEV1 and FVC were calculated for each pixel within the lung areas (16). A functional EIT image was constructed as pixel-wise FEV1, FVC, FEV1/FVC, forced inspiratory vital capacity [FIVC defined in ATS/ERS 2019 (13)], mean flow rate 25 to 75% (MEF between 25 and 75% of FVC), T75 (time required for exhaling 75% of FVC), and time constant (which is described in detail in the next paragraph).

After defining the obstructed pixels (Equation 1), “regional obstructive ratio” *rOR* was calculated based on the size of obstructed regions in relation to the entire lung area (Equations 2, 3).


(1) $$P_{n}=\{\begin{array}{c} 1,\;if\frac{FEV1_{n}}{FVC_{n}}<0.7\\ 0,\;if\;\frac{FEV1_{n}}{FVC_{n}}\geq 0.7 \end{array},\;n\in lung$$

(2) $$\begin{array}{l} A_{l}=\{\begin{array}{l} 1,\;if\;l\in lung\;\\ 0,\;otherwise \end{array} \end{array}$$

(3) $$\begin{array}{l} rOR=\frac{\sum_{n}P_{n}}{\sum_{l}A_{l}}\times 100\% \end{array}$$

The *rOR* represents the area where FEV1/FVC is smaller than 0.7. The threshold “0.7” was selected according to the definition of COPD. The higher the value, the more area shows an airway obstruction pattern. In addition, similar to the global inhomogeneity (GI) index proposed in a previous study (17), the GI of functional FEV1 and FEV1/FVC images was calculated (Equations 4).


(4) $$\begin{array}{l} GI_{FEV1}=\frac{\sum_{l\in lung}\left|{\left(FEV1\right)}_{l}-Median\left({\left(FEV1\right)}_{lung}\right)\right|}{\sum_{l\in lung}{\left(FEV1\right)}_{l}} \end{array}$$

where the *lung* was the area identified as described previously (18). *FEV1* could be substituted with pixel-wise *FEV1/FVC*. The higher the GI value, the more inhomogeneous the functional image.

Furthermore, time constant map was calculated, as described in previous studies (19–21). In brief, for every pixel within the lung region, the regional time constant was calculated by fitting the following exponential equation:


(5) $$\begin{array}{l} Z\left(t\right)=Z_{0}\cdot e^{\frac{-t}{\tau }}+c \end{array}$$

where *Z*(*t*) is the relative impedance for a pixel within the lung at time point t, *Z*_0_ is the impedance at the start of expiration, *t* represents the time from the end-inspiration to the end-expiration, τ denotes the regional time constant, and *c* the end-expiratory volume. Patients with obstructive lung disease require a longer time to empty the lungs, which thus leads to a longer time constant. Instead of calculating it for every tidal breath as proposed earlier, the time constant map (functional EIT with pixels showing τ) was calculated from the FVC data in the present study. The median of regional τ (τ_med_) and interquartile range (τ_iqr_) were calculated.

The data processing and statistical analysis were conducted using MATLAB R2023a (The MathWorks Inc., Natick, USA). The Lilliefors test was used for normality testing. Normally distributed data were expressed as mean ± standard deviation. An unpaired Student's *t*-test was used to compare the differences between spirometry- and EIT-based parameters between healthy subjects and PRISm subjects. For non-normally distribution, data were expressed as median (interquartile range). A two-sided Wilcoxon rank-sum test was used instead. Pearson's linear correlation coefficients were calculated between EIT-based parameters and spirometry-based parameters. A *p*-value of < 0.05 was considered statistically significant.

## Results

**Figure 1:**
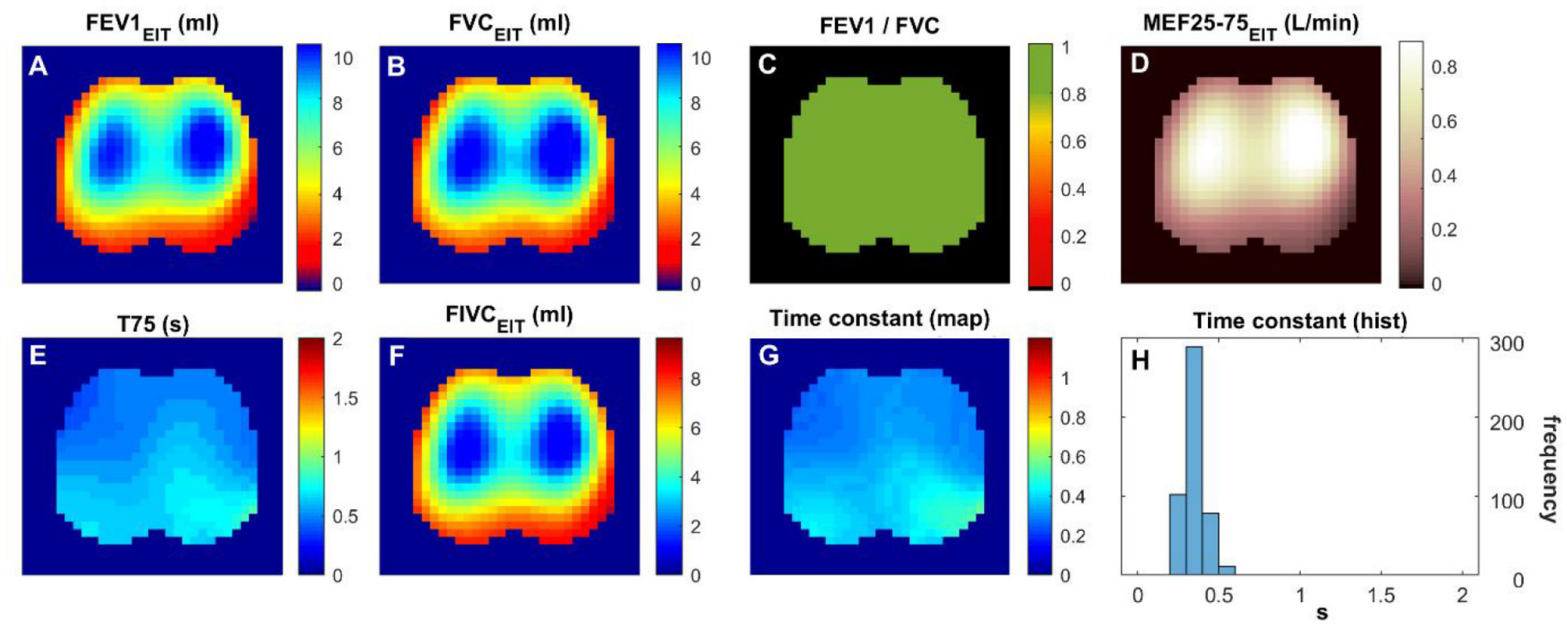
Functional EIT images visualizing the regional lung function of a healthy volunteer. **(A–H)** FEV1/FVC: the ratio of forced expiratory volume in the first second to forced vital capacity. Time constant: time related to the exponential decay of lung volume during expiration. The values in the pixels are indicated by the color bar on the right side of the sub-figures. FEV1, forced expiratory volume in the first second; FVC, forced vital capacity; FIVC, forced inspiratory vital capacity; MEF 25–75, mean flow rate between 25 and 75% of FVC; T75, time required for exhaling 75% of FVC.

**Figure 2:**
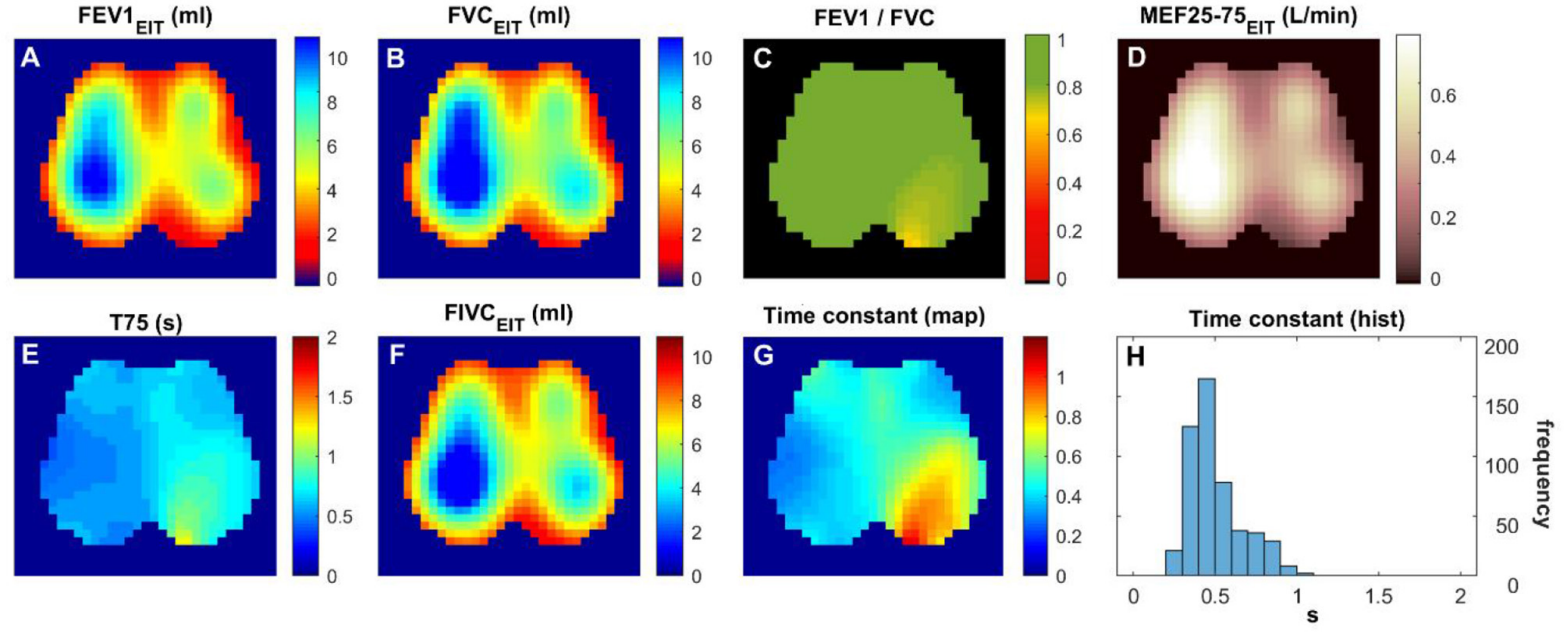
Functional EIT images visualizing the regional lung function of a subject with PRISm. **(A–H)** FEV1/FVC: the ratio of forced expiratory volume in the first second to forced vital capacity. Time constant: time related to the exponential decay of lung volume during expiration. The values in the pixels are indicated by the color bar on the right side of the sub-figures. FEV1, forced expiratory volume in the first second; FVC, forced vital capacity; FIVC, forced inspiratory vital capacity; MEF 25–75, mean flow rate between 25 and 75% of FVC; T75, time required for exhaling 75% of FVC.

A total of 21 subjects were screened, and 16 matched the PRISm definition. Subjects' demographics and their lung function measured with spirometry are summarized in Table 1. Figures 1 and 2 show the various functional EIT images visualizing the lung function parameters in one healthy subject (Figure 1) and one PRISm subject (Figure 2). The levels of FEV1, FVC, and FIVC were similar in both lungs in the healthy volunteers (Figures 1A, B and F). The ratio of FEV1/FVC was very close to 1 (as indicated in green in Figure 1C). T75 and the time constant were quite low (Figure 1E and G) and mainly distributed approximately 0.4 s (Figure 1H). On the contrary, the subject with PRISm had flow limitation mainly in his left lung, as indicated by a decrease in FEV1 (Figure 2A), FVC (Figure 2B), FEV1/FVC (Figure 2C), MEF25-75 (Figure 2D), and FIVC (Figure 2F), and an increase in T75 (Figure 2E) and time constant (Figure 2G). To be specific, the main pulmonary dysfunction was located in the left dorsal region, as highlighted in Figures 2C, E and G.

**Table 1:** Subjects' demographics and lung function measured with spirometry.

**Characteristics**	**Healthy (*n* = 16)**	**PRISm (*n* = 16)**	***p***
Men: women	14:2	14:2	1.0
Age (years)	39.3 ± 10.0	52.3 ± 18.7	**0.04**
Height (cm)	172.7 ± 6.7	170.5 ± 5.2	0.37
Weight (kg)	73.2 ± 9.7	70.4 ± 13.5	0.54
FEV1 %pred	94.6 ± 6.2	68.3 ± 11.7	**<0.0001**
FEV1/FVC	0.80 ± 0.05	0.78 ± 0.05	0.32
Smokers:non-smokers	9:7	10:6	0.72
Cigarette (pack-year)	15.0 ± 7.7	30 ± 13.9	**<0.001**

The *p* values in bold indicate statistical significance. Table shows that there are significant differences in age, FEV1 %pred, and cigarette (pack–year) between the two groups.

**Figure 3:**
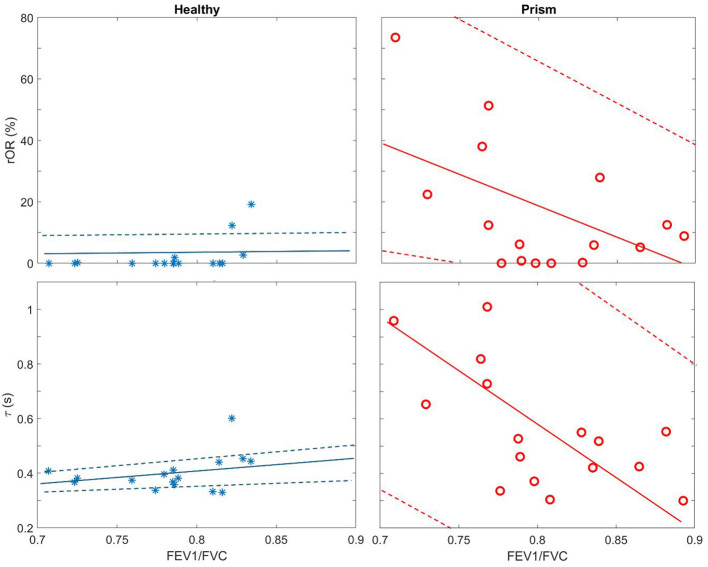
Scatter plots of regional obstructive ratio (rOR) against spirometry FEV1/FVC **(Top row)** and median of regional time constant (τ_med_) against spirometry FEV1/FVC **(Bottom row)**. Blue stars, healthy volunteers; red circles, subjects with PRISm. Solid lines are the regression lines, and dashed lines are confidence intervals.

The EIT-based parameters were compared and summarized in Table 2. All the evaluated parameters were worse in the subjects with PRISm; however, statistical significances were only found in three parameters (i.e., *rOR*, τ_med_, and τ_iqr_). EIT-based parameters were not correlated with spirometry-based parameters, except spirometry FEV1/FVC with *rOR* (*r* = −0.53, *p* = 0.03) and with τ_med_ (*r* = −0.62, *p* = 0.01) in subjects with PRISm. Spirometry FEV1/FVC against τ_med_ and *rOR* is plotted in Figure 3. In healthy volunteers, τ_med_ and *rOR* values were quite low, and, therefore, not statistically correlated with spirometry parameters.

**Table 2:** Comparison of EIT-based parameters between healthy subjects and PRISm subjects.

**Parameters**	**Healthy (*n* = 16)**	**PRISm (*n* = 16)**	**CI**	***p***
GI-FEV1	0.38 ± 0.09	0.40 ± 0.06	[−0.08, 0.02]	0.25
GI-FEV1/FVC	0.39 ± 0.09	0.42 ± 0.11	[−0.18, 0.01]	0.31
*rOR* (%)	0.00 (2.46)	7.50 (24.65)	[−27.03, 0.48]	**0.03**
T75 (s)	0.70 ± 0.14	0.85 ± 0.32	[−0.37, 0.06]	0.15
τ_med_ (s)	0.40 ± 0.10	0.56 ± 0.22	[−0.30, 0.01]	**0.03**
τ_iqr_ (s)	0.07 (0.04)	0.17 (0.35)	[−0.34, 0.01]	**0.02**

GI, global inhomogeneity index; FEV1, forced expiratory volume in the first second; FVC, forced vital capacity; *rOR*, regional obstructive ratio; T75, time required for exhaling 75% of FVC; τ_med_, median of regional time constant; τ_iqr_, interquartile range of regional time constant; CI, confidence interval for the true difference between healthy subjects and PRISm subjects. The *p* values in bold indicate statistical significance. Table indicates differences in EIT-based parameters like rOR (regional obstructive ratio, *p* = 0.03), τ_med_ (median of regional time constant, *p* = 0.03), and τ_iqr_ (interquartile range of regional time constant, *p* = 0.02) between the two groups.

## Discussion

In the present study, we demonstrated the ability of EIT-based regional lung function parameters to assess lung status in subjects with PRISm. Regional lung function differed between healthy subjects and those with PRISm. Given the small variation among healthy subjects and the full range observed in PRISm, EIT-based parameters might be more specific in detecting physiological abnormalities compared to traditional spirometry, allowing visualization and localization of the dysfunction areas. With such information, EIT might stratify PRISm subtypes (e.g., small airway vs. parenchymal disease), which was not explored in the current study. How this regional information may reform clinical practice requires further investigation.

In previous studies, regional lung function was found to be different between healthy subjects and subjects with asthma and COPD (9, 10, 22, 23). We also demonstrated the feasibility of training subjects for respiratory physiotherapy with the visual feedback provided by EIT (24, 25). Since subjects with PRISm are at risk of developing airflow obstruction over time, early and accurate identification of those at risk may help prevent disease progression. As observed in Figure 3, even in healthy volunteers, spirometry-based FEV1/FVC varied from 0.7 to 0.85, which overlaps with the range seen in PRISm. These results suggested that the specificity of spirometry in identifying PRISm might be limited. On the other hand, the variations of the EIT-based *rOR* and τ_med_ were relatively small in healthy subjects (Figure 3 left) but became large in PRISm (Figure 3 right). One dilemma is that the definition of PRISm is based on spirometry. Therefore, the superiority of specificity in PRISm identification with EIT cannot be verified. Nevertheless, EIT-based regional lung function evaluation provides various visualized functional images (Figures 1, 2) with certain spatial resolution, which helps the clinical users to identify the location of dysfunction. Currently, to the best of our knowledge, no other established techniques could provide similar information at the bedside. Some might argue that computed tomography (CT) or spirometry provides structural and functional information about the respiratory system. Indeed, we could “guess” the lung function and status with the information provided by CT scans, but it is not functional. Spirometry provides global lung function without any regional information. Forced oscillation technique could be one of the promising techniques (26). Although it distinguishes information from large airways to small airways, the location of the dysfunction is not captured.

Two previous studies examined the EIT-based regional lung function in PRISm (27, 28). While Li et al. reported spatial heterogeneity and expiratory time (T-75), they did not calculate time constants, which provide direct physiological insights into lung mechanics (e.g., compliance and resistance) (28). Similarly, Zhao et al. focused on obstructive ratios (rOR) and symptom correlation but omitted temporal parameters entirely (27). In addition, we used frequency matching for gender, height, and weight between PRISm and healthy groups, reducing confounding biases.

All the evaluated EIT-based parameters were worse in the subjects with PRISm than in the healthy subjects (Table 2). Due to the limited number of subjects in each group, only three parameters showed statistical significance. Another cause that led to this result could be the inclusion criteria for the healthy group. Although we have matched the gender, height, and weight, we did not match the age and did not exclude subjects with a smoking history. Age is a known determinant of lung function and could confound the observed differences. The current study did not match the age, which was a limitation. Up to now, no study has investigated the regional lung function in various age groups. It is unknown how age difference impacts the EIT-based regional lung function assessment. Smoking history in the control group may introduce bias. A previous study suggests that even subjects with a >10 pack-year smoking history might not show defects in spirometry, EIT-based regional lung function, or CT assessment (29). Nevertheless, we cannot rule out the potential bias, which might have contributed to statistical insignificance in some parameters. Two subjects in the healthy group showed raised *rOR*, and one subject showed raised τ_med_ (Figure 3 left), suggesting that subjects with normal spirometry might not be “normal” in EIT. Further confirmation with other techniques, e.g., CT or ultrasound, should be conducted, which was not performed in the current study. The EIT-derived parameters (especially rOR and τ_med_) are relatively new. Further research in this area is needed to validate and consolidate their clinical relevance.

In addition to the above-mentioned limitations, no sample size was calculated prior to the study. At the time of the study, no literature provided relevant information. In this preliminary pilot study, only a convenience sample was collected, which resulted in low statistical power and some parameters not reaching statistical significance. Nevertheless, this study provides a standpoint for the design of further studies that may demonstrate the clinical benefits when using regional lung function information in PRISm (or suspected PRISm). The association between EIT-based regional lung function and patient-reported outcomes regarding patient symptoms, quality of life, or exacerbation risk would strengthen clinical relevance. Unfortunately, it was not a clinical routine to complete the questionnaires, and we did not include these tools in our study design. We recognize that healthy volunteers may not fully represent the general population in addition to age and smoking history which we acknowledged. Future studies could incorporate population-based controls or inverse probability weighting.

## Conclusion

EIT-based regional lung function provides additional information to traditional spirometry, which visualizes and localizes regional lung function defects.

## Data Availability

The original contributions presented in the study are included in the article/supplementary material, further inquiries can be directed to the corresponding authors.

## References

[1] Wan ES Balte P Schwartz JE Bhatt SP Cassano PA Couper D. Association between preserved ratio impaired spirometry and clinical outcomes in US adults. JAMA. (2021) 326:2287–98. 10.1001/jama.2021.20939 34905031 PMC8672237

[2] Wijnant SRA De Roos E Kavousi M Stricker BH Terzikhan N Lahousse L. Trajectory and mortality of preserved ratio impaired spirometry: the Rotterdam study. Eur Respir J. (2020) 55:1901217. 10.1183/13993003.01217-2019 31601717

[3] Parekh TM Bhatia S Cherrington A Kim YI Lambert A Iyer A. Factors influencing decline in quality of life in smokers without airflow obstruction: the COPDGene study. Respir Med. (2020) 161:105820. 10.1016/j.rmed.2019.105820 31759270 PMC7534974

[4] Global Initiative for Chronic Obstructive Lung Disease. Global Initiative for Chronic Obstructive Lung Disease-GOLD. (2025). Available online at: https://goldcopd.org/ (Accessed September 30, 2025).

[5] Frerichs I Amato MB van Kaam AH Tingay DG Zhao Z Grychtol B. Chest electrical impedance tomography examination, data analysis, terminology, clinical use and recommendations: consensus statement of the TRanslational EIT developmeNt stuDy group. Thorax. (2017) 72:83–93. 10.1136/thoraxjnl-2016-208357 27596161 PMC5329047

[6] Frerichs I Hinz J Herrmann P Weisser G Hahn G Dudykevych T. Detection of local lung air content by electrical impedance tomography compared with electron beam CT. J Appl Physiol (1985). (2002) 93:660–6. 10.1152/japplphysiol.00081.2002 12133877

[7] Hsu HJ Chang HT Zhao Z Wang PH Zhang JH Chen YS. Positive end-expiratory pressure titration with electrical impedance tomography and pressure-volume curve: a randomized trial in moderate to severe ARDS. Physiol Meas. (2021) 42:014002. 10.1088/1361-6579/abd679 33361553

[8] Becher T Buchholz V Hassel D Meinel T Schädler D Frerichs I. Individualization of PEEP and tidal volume in ARDS patients with electrical impedance tomography: a pilot feasibility study. Ann Intensive Care. (2021) 11:89. 10.1186/s13613-021-00877-7 34080074 PMC8171998

[9] Frerichs I Zhao Z Becher T Zabel P Weiler N Vogt B. Regional lung function determined by electrical impedance tomography during bronchodilator reversibility testing in patients with asthma. Physiol Meas. (2016) 37:698–712. 10.1088/0967-3334/37/6/698 27203725

[10] Vogt B Zhao Z Zabel P Weiler N Frerichs I. Regional lung response to bronchodilator reversibility testing determined by electrical impedance tomography in chronic obstructive pulmonary disease. Am J Physiol Lung Cell Mol Physiol. (2016) 311:L8–19. 10.1152/ajplung.00463.2015 27190067

[11] Zhao Z Müller-Lisse U Frerichs I Fischer R Möller K. Regional airway obstruction in cystic fibrosis determined by electrical impedance tomography in comparison with high resolution CT. Physiol Meas. (2013) 34:N107–14. 10.1088/0967-3334/34/11/N107 24150032

[12] Munir B Murphy EK Mallick A Gutierrez H Zhang F Smith C. Electrical impedance tomography as a quantitative measure of pulmonary function in ALS patients. Neurology. (2020) 94:1187. 10.1212/WNL.94.15_supplement.1187 32240997

[13] Graham BL Steenbruggen I Miller MR Barjaktarevic IZ Cooper BG Hall GL. Standardization of spirometry 2019 update an official American thoracic society and european respiratory society technical statement. Am J Respir Crit Care Med. (2019) 200:e70–88. 10.1164/rccm.201908-1590ST 31613151 PMC6794117

[14] Krueger-Ziolek S Schullcke B Kretschmer J Müller-Lisse U Möller K Zhao Z. Positioning of electrode plane systematically influences EIT imaging. Physiol Meas. (2015) 36:1109–18. 10.1088/0967-3334/36/6/1109 26006327

[15] Yang L Gao Z Cao X Sun S Wang C Wang H. Electrical impedance tomography as a bedside assessment tool for COPD treatment during hospitalization. Front Physiol. (2024) 15:1352391. 10.3389/fphys.2024.1352391 38562620 PMC10982416

[16] Vogt B Pulletz S Elke G Zhao Z Zabel P Weiler N. Spatial and temporal heterogeneity of regional lung ventilation determined by electrical impedance tomography during pulmonary function testing. J Appl Physiol (1985). (2012). 113:1154-1161. 10.1152/japplphysiol.01630.2011 22898553

[17] Zhao Z Moller K Steinmann D Frerichs I Guttmann J. Evaluation of an electrical impedance tomography-based global inhomogeneity index for pulmonary ventilation distribution. Intensive Care Med. (2009) 35:1900–6. 10.1007/s00134-009-1589-y 19652949

[18] Zhao Z Steinmann D Müller-Zivkovic D Martin J Frerichs I Guttmann J. A lung area estimation method for analysis of ventilation inhomogeneity based on electrical impedance tomography. J Xray Sci Technol. (2010) 18:171–82. 10.3233/XST-2010-0252 20495244

[19] Karagiannidis C Waldmann AD Róka PL Schreiber T Strassmann S Windisch W. Regional expiratory time constants in severe respiratory failure estimated by electrical impedance tomography: a feasibility study. Crit Care. (2018) 22:221. 10.1186/s13054-018-2137-3 30236123 PMC6148957

[20] Pikkemaat R Tenbrock K Lehmann S Leonhardt S. Electrical impedance tomography: new diagnostic possibilities using regional time constant maps. Appl Cardiopulm Pathophysiol. (2012) 16:212–25.

[21] Strodthoff C Kähkönen T Bayford RH Becher T Frerichs I Kallio M. Bronchodilator effect on regional lung function in pediatric viral lower respiratory tract infections. Physiol Meas. (2022) 43:104001. 10.1088/1361-6579/ac9450 36137548

[22] Vogt B Löhr S Zhao Z Falkenberg C Ankermann T Weiler N. Regional lung function testing in children using electrical impedance tomography. Pediatr Pulmonol. (2018) 53:293–301. 10.1002/ppul.23912 29136345

[23] Vogt B Deuß K Hennig V Zhao Z Lautenschläger I Weiler N. Regional lung function in nonsmokers and asymptomatic current and former smokers. ERJ Open Res. (2019) 5:00240-2018. 10.1183/23120541.00240-2018 31321224 PMC6628636

[24] Li Q Li Y Niu G Li M Deng J Möller K. Chest physiotherapy guided by electrical impedance tomography in high-dependency unit patients with pulmonary diseases: an introduction of methodology and feasibility. Crit Care. (2023) 27:24. 10.1186/s13054-023-04308-w 36650565 PMC9847064

[25] Yang L Gao Z Cao X Wang C Wang H Dai J. Visualizing pursed lips breathing of patients with chronic obstructive pulmonary disease through evaluation of global and regional ventilation using electrical impedance tomography. Physiol Meas. (2024) 45:2724–41. 10.1088/1361-6579/ad33a1 38479002

[26] Tirelli C Mira S Italia M Maggioni S Intravaia C Zava M. Applications of forced oscillatory technique in obstructive and restrictive pulmonary diseases: a concise state of the art. J Clin Med. (2025) 14:5718. 10.3390/jcm14165718 40869544 PMC12386753

[27] Zhao Z Li J He R Möller K Frerichs I Ge H. Regional pulmonary function testing: a new tool for screening subjects with preserved ratio impaired spirometry? ERJ Open Res. (2025) 11:01364–2024. 10.1183/23120541.01364-2024 40630380 PMC12230744

[28] Li J Zhao Z He R Xie Y Xu Z Ni C. Regional lung function assessment using electrical impedance tomography in COPD, PRISm, and normal spirometry subjects: insights into early diagnostic potential. BMC Pulm Med. (2025) 25:215. 10.1186/s12890-025-03668-z 40325454 PMC12051352

[29] Qu S Feng E Dong D Yang L Dai M Frerichs I. Early screening of lung function by electrical impedance tomography in people with normal spirometry reveals unrecognized pathological features. Nat Commun. (2025) 16:622. 10.1038/s41467-024-55505-2 39805822 PMC11731049

